# Epidemiology and prevalence of pulmonary sequestration in Chinese population, 2010–2019

**DOI:** 10.1186/s12890-023-02308-8

**Published:** 2023-01-09

**Authors:** Yuyang Gao, Wenli Xu, Wenyan Li, Zhiyu Chen, Qi Li, Zhen Liu, Hanmin Liu, Li Dai

**Affiliations:** 1 National Center for Birth Defects Monitoring, West China Second University Hospital, Sichuan University, No.17 Section 3 Renminnanlu, Chengdu, 610041 Sichuan China; 2 Pediatric Department, The Joint Laboratory for Pulmonary Development and Related Diseases, West China Institute of Women and Children’s Health, West China Second University Hospital, Sichuan University, No.17 Section 3 Renminnanlu, Chengdu, 610041 Sichuan China; 3 National Health Commission Key Laboratory of Chronobiology, Sichuan University, Chengdu, Sichuan China; 4 Key Laboratory of Birth Defects and Related Diseases of Women and Children (Sichuan University), Ministry of Education, Chengdu, Sichuan China; 5 Med-X Center for Informatics, Sichuan University, Chengdu, Sichuan China; 6 NHC Key Laboratory of Chronobiology, Sichuan University, Chengdu, Sichuan China

**Keywords:** Pulmonary sequestration, Epidemiology, Prevalence rate, Chinese

## Abstract

**Background:**

Pulmonary sequestration (PS) is the second common congenital lung malformation and has been known for over 150 years. However, there is a scarcity of epidemiological studies on it. This study aimed to characterize the epidemiology of pulmonary sequestration in Chinese population in the recent decade by using a nationwide database.

**Methods:**

Using data from the Chinese Birth Defects Monitoring Network during 2010–2019, the prevalence rates for PS were calculated by birth year, maternal age, residence area, geographical region, and infant sex. Variations in prevalence and changes over time were further examined. Other variables of interest for analysis included the pregnancy outcomes of affected infants, the prenatal diagnosis, and the co-occurring anomalies of PS cases.

**Results:**

During the study period, we identified an average prevalence rate of 0.31, 0.11, and 0.42 per 10,000 live and still births for the isolated, non-isolated, and overall PS, respectively. An upward trend was observed for each category of PS. The prevalence rates varied significantly by maternal age (< 20 years, 0.34/10,000; 20–24 years, 0.33/10,000; 25–29 years, 0.45/10,000; 30–34 years, 0.46/10,000; ≥ 35 years, 0.36/10,000), residence area (urban vs. rural, 0.51/10,000 vs. 0.30/10,000), geographical region (western, 0.33/10,000; eastern, 0.49/10,000; central, 0.43/10,000), and by infant sex (male vs. female, 0.45/10,000 vs. 0.38/10,000). Non-isolated PS cases were more likely born prematurely than isolated cases (15.29% vs. 7.83%). 40.28% and 33.80% of non-isolated cases were accompanied by additional respiratory, and circulatory system malformations, respectively.

**Conclusions:**

The study presents for the first time the prevalence of pulmonary sequestration in Chinese population. The rising prevalence and relatively poor perinatal outcome of affected fetuses or newborns indicate the necessity to improve perinatal management of PS.

## Introduction

Pulmonary sequestration (PS) is a rare congenital malformation characterized by a non-functional mass of lung tissue that does not communicate with the tracheobronchial tree and receives an anomalous vascular blood supply from the systemic circulation [1]. The most widely accepted theory is that PS results from the formation of an accessory lung bud below the normal lung buds [2]. It may occur as an isolated lesion or in association with other malformations [2–4]. Clinically, PS cases are usually divided into the intralobar and extralobar types. Most children affected by PS have a good prognosis, some of them may experience a high risk of infection, hemoptysis, hemorrhage, and heart failure in rare cases [5–7]. The etiology of PS remains poorly understood. Epidemiology study is of high importance to identify the distribution of disorders, risk factors, underlying mechanisms, and evidence for making preventive measures.

There is a scarcity of epidemiological study on PS, though it ranks the second most common congenital lung malformations (CLM) [8]. Published studies indicate that PS accounts for 0.15–8.3% of pulmonary anomalies [9, 10]. Nearly all of them are case studies, mainly focusing on the clinical manifestations, diagnosis, treatment, and prognosis [11–14], reliable prevalence data on PS are still lacking [2, 15]. To characterize its epidemiology in Chinese population, we used data from the Chinese Birth Defects Monitoring Network (CBDMN) during 2010–2019 to analyze the prevalence pattern, with special interests in secular trends, perinatal outcomes, and associated malformations with non-isolated cases.

## Methods

### Study subjects

The study population included live births, stillbirths, and termination of pregnancies (TOP) with PS that were identified by CBDMN during 2010–2019. CBDMN is a nationwide hospital-based birth defects surveillance system that consists of 763 member hospitals and covers more than 10% of annual births in China. The details on case ascertainment, data collection, and quality control of this system have been published elsewhere [16–20]. Briefly, the pediatric, obstetric, or ultrasound experts in member hospitals are responsible for the diagnosis of birth defect cases during the gestation and within 7 days after birth. Monthly summary data on live births or stillbirths with gestational age ≥ 28 weeks, and information of birth defects cases regardless of gestational age are regularly collected and reported by trained surveillance staff through an online data reporting system. All anomalies in CBDMN database are finally coded by a national panel according to the International Classification of Disease 10th version (ICD-10). Particularly, records with the ICD-10 code for PS (Q33.2) were extracted from CBDMN database for the current analysis, including records of isolated (PS only) and non-isolated cases (accompanied by other major pulmonary or non-pulmonary congenital anomalies).

### PS diagnostics

The most commonly used method for diagnosing PS is ultrasonography. The presence of an echogenic fetal/infant thoracic mass and an aberrant systemic arterial supply by color-flow Doppler are the critical criteria of PS diagnosis [21–23]. Other techniques include computer tomography (CT), and magnetic resonance imaging (MRI) [24]. In the surveillance system, the diagnosis of birth defects is required to be carried out following CBDMN surveillance manual and related clinical guidelines. According to the Administrative Method on Antenatal Diagnostic Techniques Regulation issued by China Health Commission in 2003, the prenatal diagnosis should be made by at least two qualified doctors in a certified prenatal diagnosis center, and the antenatally diagnosed cases should be confirmed after birth [16].

This research was approved by the Medical Ethics Committee of the West China Second University Hospital of Sichuan University. All methods were carried out in accordance with relevant guidelines and regulations or declaration of Helsinki. And the individual informed consent was waived.

### Statistical analysis

The prevalence rate (PR) was expressed as the number of PS cases per 10,000 live and still perinatal births. Prevalence analysis was conducted according to the following categories: birth year (2010–2019), maternal age (< 20, 20–24, 25–29, 30–34, and ≥ 35 years), residence (urban and rural), geographic region (eastern, central, and western), and infant sex (female and male). The maternal residence was classified into urban (cities or urbanized areas or neighborhood committee) and rural areas (villages or countryside), depending on the last place the mother resided for at least 1 year [20]. The geographic region was divided by geographical locations and economic conditions [25, 26]. The distributions of birth weight (< 2500 g, 2500–3999 g, and ≥ 4000 g), gestational age (< 37, 37–41, and ≥ 42 weeks of gestation), and pregnancy outcomes (stillbirth, early neonatal death, alive more than 7 days) were compared between isolated and non-isolated cases. Chi-square test was used to examine differences in rates or percentages between groups, and linear Chi-square test was adopted to detect prevalence changes over time. Poisson regression was applied for estimating the adjusted prevalence rate ratios (PRR). Statistical analyses were performed by R 3.5.3 (R Development Core Team 2019). The statistical significance level for α was set at 0.05.

## Results

**Fig. 1:**
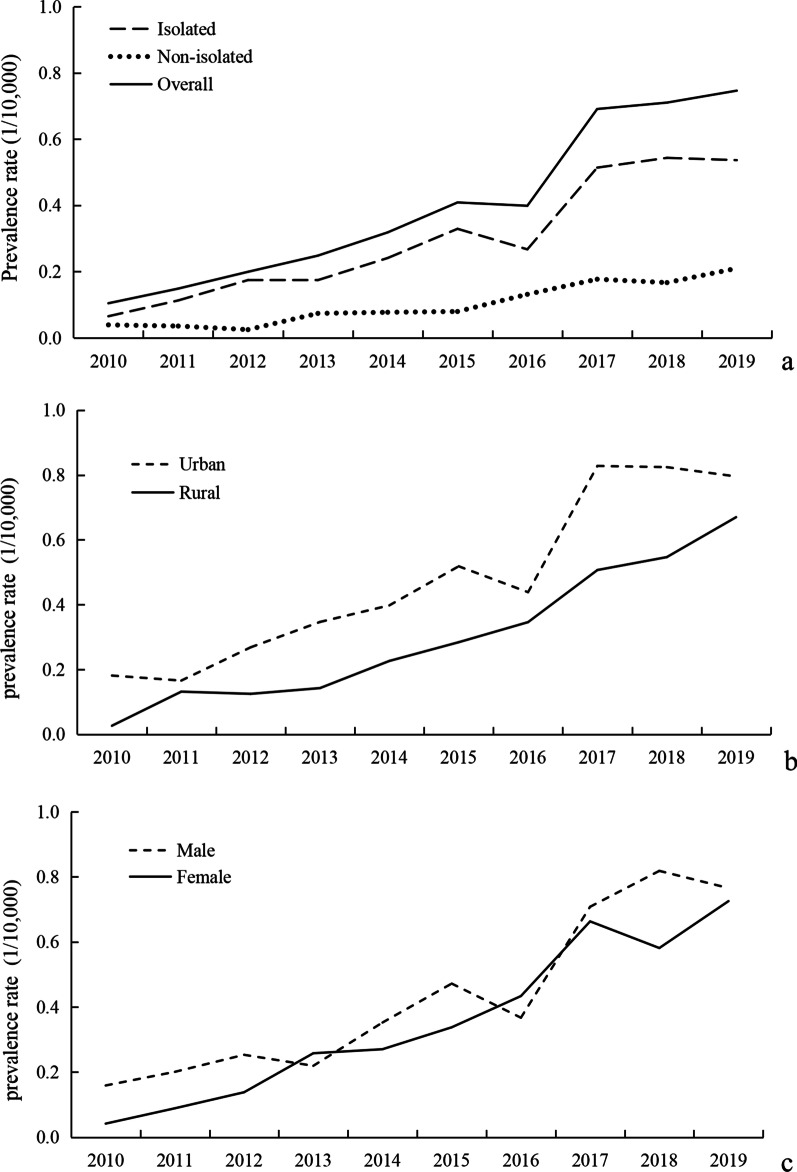
Time trends in the prevalence of pulmonary sequestration in China, 2010–2019. **a** Stratified by case type (overall, χ^2^ = 229.73, *p* < 0.001; isolated, χ^2^ = 164.08, *p* < 0.001; non-isolated, χ^2^ = 65.90, *p* < 0.001), **b** stratified by maternal residence (urban, χ^2^ = 23.64, *p* < 0.001; rural, χ^2^ = 41.04, *p* < 0.001), and **c** stratified by sex (male, χ^2^ = 28.83, *p* < 0.001; female, χ^2^ = 36.83, *p* < 0.001)

Table 1 shows the prevalence rates of PS by birth year, maternal age, residence, geographical region, and infant sex. During 2010 to 2019, a total of 841 PS cases (625 isolated and 216 non-isolated cases) were identified among 20,183,999 births, yielding a prevalence rate of 0.42 (95% CI 0.39–0.45), 0.31 (95% CI 0.29–0.34), and 0.11 (95% CI 0.09–0.12) per 10,000 live and still births for the overall, isolated, and non-isolated PS, respectively. The prevalence rates of PS have been increasing substantially over the past 10 years, from 0.10 to 0.75 per 10,000 for the overall, from 0.07 to 0.54 per 10,000 for the isolated, and from 0.04 to 0.21 per 10,000 births for the non-isolated PS. When analyzing secular trends by maternal residence and infant sex, a similar increasing trend in annual prevalence rates was found (Fig. 1).

**Table 1:** Prevalence rates of pulmonary sequestration (1/10,000) in China during 2010–2019

	Birth number	Isolated PS (N = 625)			Non-isolated PS (N = 216)			Overall PS (N = 841)		
		N	PR(95%CI)	aPRR(95%CI)	N	PR(95%CI)	aPRR(95%CI)	N	PR(95%CI)	aPRR(95%CI)
*Birth year*										
2010	1,531,143	10	0.07(0.03–0.12)	1.00(ref)	6	0.04(0.01–0.09)	1.00(ref)	16	0.10(0.06–0.17)	1.00(ref)
2011	1,681,096	19	0.11(0.07–0.18)	1.73(0.82–3.87)	6	0.04(0.01–0.08)	0.91(0.29–2.92)	25	0.15(0.10–0.22)	1.42(0.77–2.72)
2012	2,005,526	35	0.18(0.12–0.24)	**2.65(1.36–5.65)**	5	0.02(0.01–0.06)	0.63(0.18–2.09)	40	0.20(0.14–0.27)	**1.89(1.08–3.47)**
2013	1,893,560	33	0.17(0.12–0.25)	**2.58(1.32–5.54)**	14	0.07(0.04–0.12)	1.74(0.69–4.97)	47	0.25(0.18–0.33)	**2.27(1.31–4.14)**
2014	2,198,802	53	0.24(0.18–0.32)	**3.57(1.90–7.46)**	17	0.08(0.05–0.12)	1.94(0.80–5.37)	70	0.32(0.25–0.40)	**2.96(1.76–5.28)**
2015	1,883,843	62	0.33(0.25–0.42)	**5.02(2.70–10.42)**	15	0.08(0.04–0.13)	2.03(0.82–5.69)	77	0.41(0.32–0.51)	**3.90(2.34–6.93)**
2016	2,432,979	65	0.27(0.21–0.34)	**4.03(2.17–8.34)**	32	0.13(0.09–0.19)	**3.30(1.48–8.76)**	97	0.40(0.32–0.49)	**3.75(2.28–6.61)**
2017	2,315,621	119	0.51(0.43–0.62)	**7.78(4.29–15.86)**	41	0.18(0.13–0.24)	**4.56(2.09–11.97)**	160	0.69(0.59–0.81)	**6.57(4.06–11.44)**
2018	2,097,800	114	0.54(0.45–0.65)	**8.20(4.52–16.74)**	35	0.17(0.12–0.23)	**4.24(1.92–11.22)**	149	0.71(0.60–0.83)	**6.72(4.14–11.71)**
2019	2,143,629	115	0.54(0.44–0.64)	**8.12(4.47–16.56)**	45	0.21(0.15–0.28)	**5.25(2.42–13.75)**	160	0.75(0.64–0.87)	**7.04(4.35–12.26)**
*Maternal age (years)*										
< 20	438,500	13	0.30(0.16–0.51)	1.19(0.64–1.99)	2	0.46(0.01–0.17)	0.49(0.08–1.54)	15	0.34(0.19–0.56)	1.00(0.57–1.61)
20–24	3,968,592	96	0.24(0.20–0.30)	0.94(0.74–1.18)	33	0.08(0.06–0.12)	0.88(0.58–1.29)	129	0.33(0.27–0.39)	0.92(0.75–1.13)
25–29	8,403,602	278	0.33(0.29–0.37)	1.00(ref)	103	0.12(0.10–0.15)	1.00(ref)	381	0.45(0.41–0.50)	1.00(ref)
30–34	5,032,691	167	0.33(0.28–0.39)	0.89(0.73–1.08)	64	0.13(0.10–0.16)	0.93(0.68–1.27)	231	0.46(0.40–0.52)	0.90(0.76–1.06)
≥ 35	2,340,614	71	0.30(0.24–0.38)	0.78(0.60–1.01)	14	0.06(0.03–0.10)	**0.42(0.23–0.72)**	85	0.36(0.29–0.45)	**0.69(0.54–0.87)**
*Residential area*										
Rural	9,089,196	206	0.23(0.20–0.26)	1.00(ref)	69	0.08(0.06–0.10)	1.00(ref)	275	0.30(0.27–0.34)	1.00(ref)
Urban	11,094,803	419	0.38(0.34–0.42)	**1.54(1.30–1.83)**	147	0.13(0.11–0.16)	**1.65(1.24–2.23)**	566	0.51(0.47–0.55)	**1.57(1.35–1.82)**
*Geographical region*										
Western	6,263,933	147	0.23(0.20–0.28)	1.00 (ref)	58	0.09(0.07–0.12)	1.00(ref)	205	0.33(0.28–0.38)	1.00(ref)
Eastern	6,438,929	247	0.38(0.34–0.43)	**1.62(1.32–1.99)**	67	0.10(0.08–0.13)	1.10(0.77–1.56)	314	0.49(0.44–0.54)	**1.47(1.23–1.75)**
Central	7,481,137	231	0.31(0.27–0.35)	**1.33(1.09–1.64)**	91	0.12(0.10–0.15)	1.32(0.95–1.85)	322	0.43(0.38–0.48)	**1.33 (1.12–1.59)**
*Infant sex*										
Female	9,524,584	271	0.29(0.25–0.32)	1.00(ref)	88	0.09(0.07–0.11)	1.00(ref)	359	0.38(0.34–0.42)	1.00(ref)
Male	10,655,756	350	0.33(0.30–0.37)	1.16(0.99–1.36)	127	0.12(0.10–0.14)	1.30(0.99–1.71)	477	0.45(0.41–0.49)	**1.20(1.04–1.37)**

*N*, number; *PS*, pulmonary sequestration; *PR*, prevalence rate; *aPRR*, adjusted prevalence rate ratio

Adjusted for birth year, maternal age, residential area, geographical region, and infant sex

Numbers marked in bold indicate a statistically significant difference

There were statistically significant variations in the prevalence of PS by maternal age, residence, geographical region, and infant sex. With adjustment for birth year, maternal residence, infant sex, and geographical region, the lowest PRR was found in the maternal age group of ≥ 35 years (adjusted PRR: 0.69, 95% CI: 0.54–0.87). Similar maternal age-specific prevalence pattern was detected for non-isolated but not for isolated lesions. Urban PS prevalence rates were higher than rural rates (overall: 0.51/10,000 vs. 0.30/10,000; isolated: 0.38/10,000 vs. 0.23/10,000; non-isolated: 0.13/10,000 vs. 0.08/10,000), and these urban–rural disparities were further confirmed by Poisson regression analysis. There seemed no significant geographical difference in the crude prevalence of PS, but Poisson regression analysis revealed a higher adjusted PRR in eastern or central regions as compared to the western region. Male crude prevalence of PS seemed higher than the female’s, but the sex difference remained significantly only for overall PS after adjusting for birth year, maternal age and residence (male vs. female, 0.45/10,000 vs. 0.38/10,000; adjusted PRR 1.20, 95% CI 1.05–1.37).

A total of 759 cases (90.25%) were diagnosed prenatally with a mean gestational age at diagnosis of 26.51 ± 5.25 weeks. TOPs and stillbirths were collectively counted as stillbirths in CBDMN, accounting for 50.42% of all PS cases. The percentage of stillbirths in non-isolated (60.65%) was higher than that in isolated (46.88%) cases (χ^2^ = 12.17, *P* < 0.001). Nearly half of PS cases (420/841) were aborted due to antenatally diagnosed birth defects. The percentage of TOPs decreased from 56.25 to 43.75% during 2010–2019 (χ^2^ = 12.34, *P* < 0.001), with higher rate found in rural (60.00%) than in urban areas (45.05%) (χ^2^ = 16.54, *P* < 0.001).

Table 2 shows the distributions of birthweight, gestational age and perinatal outcomes of live births with PS. Totally, 9.35% of PS cases were preterm births, 6.47% were born with low birth weight, and 2.16% died within 7 days after birth. Compared to isolated cases, much more non-isolated cases tended to be born prematurely (15.29% vs. 7.83%; χ^2^ = 8.67, *P* < 0.05). No statistically significant difference was found in the birth weight and neonatal outcome distributions between isolated and non-isolated cases.

**Table 2:** Characteristics of 417 live birth cases with pulmonary sequestration

	Isolated PS cases	Non-isolated PS cases	Total PS cases
	(N = 332)	(N = 85)	(N = 417)
Gestational age (weeks)*			
< 37	26 (7.83%)	13 (15.29%)	39 (9.35%)
37–41	305 (91.87%)	70 (82.35%)	375 (89.93%)
≥ 42	1 (0.30%)	2 (2.35%)	3 (0.72%)
*Birth weight (g)*			
< 2500	19 (5.72%)	8 (9.41%)	27 (6.47%)
2500–3999	292 (87.95%)	74 (87.06%)	366 (87.77%)
≥ 4000	21 (6.33%)	3 (3.53%)	24 (5.76%)
*Early neonate outcome*			
Early neonate death	6 (1.81%)	3 (3.53%)	9 (2.16%)
Alive within 7 days	326 (98.19%)	82 (96.47%)	408 (97.84%)

*PS*, pulmonary sequestration

*χ^2^ = 8.67, *P* = 0.013

As shown in Table 3, a total of 216 cases (25.68%) were accompanied by additional chromosomal or structural malformations. The most common congenital anomalies seen in non-isolated PS cases by system included respiratory system anomalies (40.28%), circulatory system malformations (33.80%), and musculoskeletal anomalies (10.65%). Specifically, congenital pulmonary airway malformation (CPAM), congenital heart defects (CHD), and congenital diaphragmatic hernia (CDH) accounted for 93.10%, 95.89%, and 56.52% of each group of coexisting anomalies mentioned above. PS cases rarely occurred with anomalies of genital organs (0.46%), and chromosomal abnormalities (1.85%).

**Table 3:** Abnormalities associated with pulmonary sequestration

System/abnormalities	ICD-10	Cases	Percent
Nervous system	Q00-Q07	10	4.63
Hydrocephalus	Q03	7	3.24
*Eye, ear, face and neck*	Q10-Q18	6	2.78
Congenital malformations of eyelid, lacrimal apparatus and orbit	Q10	2	0.93
Malformations of ear	Q17	2	0.93
*Circulatory system*	Q20-Q28	73	33.80
Congenital heart disease	Q20-Q26	70	32.41
Malformations of cardiac septa	Q21	41	18.98
Iincluding: atrial septal defect	Q21.1	24	11.11
Ventricular septal defect	Q21.0	16	7.41
Congenital malformations of great arteries	Q25	21	9.72
Iincluding: patent ductus arteriosus	Q25.0	17	7.87
Other congenital malformations of heart	Q24	17	7.87
Malformations of cardiac chambers and connections	Q20	7	3.24
Congenital malformations of pulmonary and tricuspid valves	Q22	6	2.78
Other congenital malformations of peripheral vascular system	Q27	5	2.31
*Respiratory system*	Q30-Q34	87	40.28
Congenital malformations of lung	Q33	86	39.81
Including: congenital pulmonary airway malformation	Q33.0	81	37.50
*Cleft lip and cleft palate*	Q35-Q37	4	1.85
Cleft palate with cleft lip	Q37	3	1.39
*Digestive system*	Q38-Q45	11	5.09
Congenital malformations of gallbladder, bile ducts and liver	Q44	5	2.31
Other congenital malformations of intestine	Q43	3	1.39
Other congenital malformations of upper alimentary tract	Q40	2	0.93
*Genital organs*	Q50-Q56	1	0.46
Hypospadias	Q54	1	0.46
*Urinary system*	Q60-Q64	13	6.02
Malformations of renal pelvis and ureter	Q62	7	3.24
Cystic kidney disease	Q61	6	2.78
Other malformations of kidney	Q63	2	0.93
*Musculoskeletal system*	Q65-Q79	23	10.65
Other malformations of musculoskeletal system	Q79	17	7.87
Including: congenital diaphragmatic hernia	Q79.0	13	6.02
Deformities of feet	Q66	2	0.93
Polydactyly	Q69	2	0.93
Other congenital malformations of limb(s)	Q74	2	0.93
*Chromosomal abnormalities*	Q90-Q99	4	1.85
Other chromosome abnormalities, not elsewhere classified	Q99	3	1.39
Down’s syndrome	Q90	1	0.46
*Other malformations*	Q80-Q89	7	3.24
Other congenital malformations, not elsewhere classified	Q89	7	3.24

## Discussion

This descriptive epidemiological study revealed that the overall prevalence rate of pulmonary sequestration in Chinese population increased from 0.10 to 0.75 per 10,000 births during 2010–2019, with an average of 0.42/10,000. PS has been known for over 150 years, and accounted for up to 8.3% of all CLM [10], but reliable prevalence data are still lacking. The reported cumulative incidence of CLM ranged from 3.0 to 4.2 per 10,000 individuals [27, 28]. Thus, the average rate of 0.42/10,000 in this study was very close to existing estimates. The increasing prevalence most likely represents the improved detection of PS in prenatal period. In the current study, nearly all PS cases were identified by ultrasonography. Similar phenomena have been observed in other CLMs like CPAM whose prevalence is heavily influenced by the wide use of ultrasound imaging in prenatal care [28, 29]. There is indeed a worldwide increase in the prevalence of CLMs that can be largely ascribed to universal availability and advances in the antenatal ultrasonography, CT, and MRI technology [13, 29, 30]. Considering that some infants with small lesions are asymptomatic and may not be discovered within 7 days after birth [8], the mean PS prevalence in the last three years in this study could represent a reliable and stable PS estimate in Chinese, but it might be still underestimated. However, the results may well reflect the current status of PS in Chinese newborns, as the nationally representative data with wide geographic and socioeconomic coverages were used.

After adjusting for potential confounders, the PS prevalence varied significantly by maternal age. A significant lower rate in ≥ 35 years maternal age group was found for the overall and non-isolated PS as compared to 25–29 years maternal age group. A review suggested no association of maternal age with fetal PS [29]. This controversy needs to be further investigated in future studies. The urban–rural disparities in PS prevalence were considerable. It may be partly explained by differences in the diagnostic capacity, environmental exposures, and socioeconomic levels between urban and rural residents. As documented in early studies, urban women in China have better socioeconomic status and health care services than rural women [20, 31], which may cause a higher detection rate of PS in urban areas. The adjusted prevalence of PS in western region was lower than those in eastern or central regions, which could be explained partly by disparities in socioeconomic status and levels of diagnosis [25, 32]. The male excess in PS was consistent with previous case studies [13, 33–35], but the underlying causes are unclear. Developmental factors, genetic components, and environmental exposures have been reported to be involved in the pathogenesis of PS [36]. More etiological and analytical epidemiological studies are needed to elucidate these differences.

In the current study, prenatally diagnosed PS accounted for 90.25% of all cases. This percentage is slightly higher than that reported in France (87%) [8], but lower than those found in several case studies with a small sample size [14, 37]. Along with improvements in prenatal ultrasonography technology, many fetal structure malformations with typical sonographic features like PS can be detected antenatally. The mean gestational age at diagnosis of prenatally diagnosed cases in the present study (26.51 ± 5.25 weeks) is significantly higher than those reported in Switzerland (22.2 ± 3.1 weeks) [35]and Korea (23.5 ± 2.2 weeks) [38]. The percentage of stillbirths in the study was as high as 50.42%, which can be ascribed to the high percentage of antenatally diagnosed cases and subsequent TOPs. Moreover, the differences in the percentage of stillbirths between urban and rural groups may reflect the marked urban–rural disparities in prenatal health care services. Although the percentage of prenatally diagnosed cases of PS in this study seems higher than some figures reported in developed countries, there is still a gap in the quality of prenatal diagnosis and subsequent management between China and developed countries that undoubtedly needs to be addressed by clinicians, public health workers, and policymakers in obstetric and perinatal health care practice.

The majority of fetuses or infants affected by PS usually have a good prognosis [35, 39]. Zhang et al. reported that the survival rate in prenatally diagnosed cases was up to 100% [34]. A small number of PS fetuses with some complications such as hydrops, lung hypoplasia, major malformation, and large pleural effusion may have a high risk of mortality [35]. Effective procedures such as maternal steroids, thoracoamniotic shunt, ex utero intrapartum treatment (EXIT) provide a good solution for this situation [29]. However, 49.94% of fetuses with PS were aborted in our study. In China, termination of pregnancy due to prenatally diagnosed anomalies is legally permitted and mainly depends on the pregnant woman’s decision. Given the favorable prognosis of PS, doctors should help pregnant women to make proper decisions by offering timely counseling, health education, and perinatal management. Of those live births with PS, 2.6% died within the first 7 days after birth, with a non-significant higher rate for non-isolated PS. Moreover, the preterm birth rate of non-isolated cases was higher than that of isolated PS. These findings indicate that co-occurring anomalies might increase the risk of some adverse perinatal outcomes like premature birth or early neonatal death.

In the current study, 25.68% PS cases were accompanied by additional malformations, highly comparable to the figure of 20.8% summarized in a review based on 540 cases [9]. Previous studies reported that 6–28.6% of PS cases occurred in association with other malformations, including CPAMs, CDH, CHD, etc. [8, 13, 15, 33, 35, 40, 41]. In our sample, the top 3 frequently coexisting anomalies of PS counted according to system were congenital respiratory system malformations (40.28%), circulatory system malformations (33.80%), and musculoskeletal anomalies (10.65%). It is noteworthy that there were 37.50%, 32.41%, and 6.02% of non-isolated PS cases were associated with CPAMs, CHDs, and CDH, respectively. Several investigations documented that the mixed-type of PS with CPAM was quite common [34, 37, 42]. It accounted for up to 50% of PS cases according to Conran RM and Stocker JT’s report [42], suggesting that these two CLMs may have a shared embryological origin [37]. More than 90% of concomitant circulatory system malformations of PS were congenital heart defects. One possible explanation is that an intrathoracic mass may compress the fetal heart and cause structural changes [38]. Consistent with previous reports [2, 6, 8, 14, 43], CDH was another frequently co-occurring malformation of PS. In our study, 1.55% of PS cases were concomitant with CDH, representing 6.02% of non-isolated cases. A cohort study reported that 3.4% of CDH patients had a pulmonary sequestration [43]. The PS-CDH association can be partly attributed to the mechanically interfering with diaphragm fusion and pleuroperitoneal canal closure at about 10 weeks of gestation [44]. Our findings indicate that a broad spectrum of congenital disorders may co-occur with PS, that need to be further investigated since associated anomalies may function as important predictors of diagnosis and prognosis.

Our study has several strengths. Firstly, the data used in this study, including the large number of PS cases identified from more than 20 million births, can ensure relatively reliable prevalence estimates of PS. Secondly, we addressed the prenatal diagnosis and subsequent management of pregnancy with PS on a nationwide scale, and found considerable urban–rural disparities in stillbirths, which provides useful evidence for future health policy-making. There are also some limitations in the current study. PS cases were diagnosed by imaging, not by pathology—the gold standard for the final diagnosis. As most PS cases remain asymptomatic after birth, intrusive pathological diagnosis can hardly be obtained in a large surveillance system like CBDMN. Some comparative studies have revealed a high concordance (> 90%) between prenatal ultrasound diagnosis and postoperative pathological confirmation [4, 45]. For the past 30 years, sonographic technology has been widely used in China for the prenatal and postnatal detection of structural malformations, including PS [23, 46]. The potential for misclassification and underestimation related to diagnosis in this study is likely to have minimal impact on the results. Also the prevalence and outcomes cannot be analyzed according to clinical PS subtypes such as extralobar and intralobar sequestration due to the data limits of CBDMN. Infants born in the member hospitals were only followed up to 7th day after birth, therefore, some minor, asymptomatic PS cases or complex syndromes might be missed, especially in rural areas. Another limitation is that there could be an underestimate of the associated chromosomal aberrations or visceral malformations with non-isolated cases because of the small number of chromosome tests and autopsy in stillbirths.

## Conclusions

Based on the nationally representative CBDMN data during 2010–2019, we for the first time presented the prevalence of pulmonary sequestration in Chinese population. The rising prevalence and relatively poor pregnancy outcome of affected fetuses or newborns indicate the necessity to improve perinatal management of PS.

## Data Availability

The data in this study were obtained from the Chinese Birth Defects Monitoring Network (CBDMN), which is co-established by the National Health Commission of the People’s Republic of China and Sichuan University. The data used in this study are owned by National Health Commission of the People’s Republic of China, and the researchers did not obtain consent to publicly share these data. However, the identified dataset is available to interested researchers upon request. For data requests, please contact the corresponding author to apply for authorization, at: daili@scu.edu.cn.

## Abbreviations

PS: Pulmonary sequestration
CBDMN: Chinese Birth Defects Monitoring Network
CLM: Congenital lung malformations
TOP: Termination of pregnancies
ICD-10: International Classification of Disease, 10th version
CT: Computer tomography
MRI: Magnetic resonance imaging
PR: Prevalence rate
PRR: Prevalence rate ratio
aPRR: Adjusted prevalence rate ratio
CPAM: Congenital pulmonary airway malformation
CHD: Congenital heart defects
CDH: Congenital diaphragmatic hernia
EXIT: Ex utero intrapartum treatment

## References

[1] Landing BH, Dixon LG (1979). Congenital malformations and genetic disorders of the respiratory tract (larynx, trachea, bronchi, and lungs). Am Rev Respir Dis.

[2] Corbett HJ, Humphrey GM (2004). Pulmonary sequestration. Paediatr Respir Rev.

[3] Correia-Pinto J, Gonzaga S, Huang Y, Rottier R (2010). Congenital lung lesions–underlying molecular mechanisms. Semin Pediatr Surg.

[4] Xu G, Zhou J, Zeng S, Zhang M, Ouyang Z, Zhao Y (2019). Prenatal diagnosis of fetal intraabdominal extralobar pulmonary sequestration: a 12-year 3-center experience in China. Sci Rep.

[5] Hall NJ, Stanton MP (2017). Long-term outcomes of congenital lung malformations. Semin Pediatr Surg.

[6] Lee BS, Kim JT, Kim EA, Kim KS, Pi SY, Sung KB (2008). Neonatal pulmonary sequestration: clinical experience with transumbilical arterial embolization. Pediatr Pulmonol.

[7] Caldeira I, Fernandes-Silva H, Machado-Costa D, Correia-Pinto J, Moura RS (2021). Developmental pathways underlying lung development and congenital lung disorders. Cells.

[8] Khen-Dunlop N, Farmakis K, Berteloot L, Gobbo F, Stirnemann J, De Blic J (2018). Bronchopulmonary sequestrations in a paediatric centre: ongoing practices and debated management. Eur J Cardio-Thorac Surg Off J Eur Assoc Cardio-Thorac Surg.

[9] Savic B, Birtel FJ, Tholen W, Funke HD, Knoche R (1979). Lung sequestration: report of seven cases and review of 540 published cases. Thorax.

[10] Coman C, Stan A, Georgescu G, Dobrinov H, Dimitriu M (1973). Present problems of intra- and extra-lobar pulmonary sequestration. Poumon Coeur.

[11] Sun X, Xiao Y (2015). Pulmonary sequestration in adult patients: a retrospective study. Eur J Cardio-Thorac Surg Off J Eur Assoc Cardio-Thorac Surg.

[12] Wei Y, Li F (2011). Pulmonary sequestration: a retrospective analysis of 2625 cases in China. Eur J Cardio-Thorac Surg Off J Eur Assoc Cardio Thorac Surg.

[13] Zhang N, Zeng Q, Chen C, Yu J, Zhang X (2019). Distribution, diagnosis, and treatment of pulmonary sequestration: report of 208 cases. J Pediatr Surg.

[14] Yoon HM, Kim EA, Chung SH, Kim SO, Jung AY, Cho YA (2017). Extralobar pulmonary sequestration in neonates: the natural course and predictive factors associated with spontaneous regression. Eur Radiol.

[15] Nunes C, Pereira I, Araújo C, Santo SF, Carvalho RM, Melo A (2015). Fetal bronchopulmonary malformations. J Mater Fetal Neonatal Med Off J Eur Assoc Perinat Med Fed Asia Ocean Perinat Soc Int Soc Perinat Obstet.

[16] Dai L, Zhu J, Liang J, Wang YP, Wang H, Mao M (2011). Birth defects surveillance in China. World J Pediatr WJP.

[17] Deng K, Qiu J, Dai L, Yi L, Deng C, Mu Y (2014). Perinatal mortality in pregnancies with omphalocele: data from the Chinese national birth defects monitoring network, 1996–2006. BMC Pediatr.

[18] Zhu J, Li X, Wang Y, Mu D, Dai L, Zhou G (2012). Prevalence of neural tube defect pregnancies in China and the impact of gestational age of the births from 2006 to 2008: a hospital-based study. J Mater Fetal Neonatal Med Off J Eur Assoc Perinat Med Fed Asia Ocean Perinat Soc Int Soc Perinat Obstet.

[19] Dai L, Zhu J, Mao M, Li Y, Deng Y, Wang Y (2010). Time trends in oral clefts in Chinese newborns: data from the Chinese national birth defects monitoring network. Birth Defects Res A.

[20] Li X, Zhu J, Wang Y, Mu D, Dai L, Zhou G (2013). Geographic and urban-rural disparities in the total prevalence of neural tube defects and their subtypes during 2006–2008 in China: a study using the hospital-based birth defects surveillance system. BMC Public Health.

[21] Becmeur F, Horta-Geraud P, Donato L, Sauvage P (1998). Pulmonary sequestrations: prenatal ultrasound diagnosis, treatment, and outcome. J Pediatr Surg.

[22] Hung JH, Shen SH, Guo WY, Chen CY, Chao KC, Yang MJ (2008). Prenatal diagnosis of pulmonary sequestration by ultrasound and magnetic resonance imaging. J Chinese Med Assoc JCMA.

[23] Chowdhury MM, Chakraborty S (2015). Imaging of congenital lung malformations. Semin Pediatr Surg.

[24] Gabelloni M, Faggioni L, Accogli S, Aringhieri G, Neri E (2021). Pulmonary sequestration: what the radiologist should know. Clin Imaging.

[25] Li Y, Mao M, Dai L, Li K, Li X, Zhou G (2012). Time trends and geographic variations in the prevalence of hypospadias in China. Birth Defects Res A.

[26] Yu X, He C, Wang Y, Kang L, Miao L, Chen J (2021). Preterm neonatal mortality in China during 2009–2018: a retrospective study. PLoS ONE.

[27] Andrade CF, Ferreira HP, Fischer GB (2011). Congenital lung malformations. J Bras Pneumol Publ Of Soc Bras Pneumol Tisilogia.

[28] Stocker LJ, Wellesley DG, Stanton MP, Parasuraman R, Howe DT (2015). The increasing incidence of foetal echogenic congenital lung malformations: an observational study. Prenat Diagn.

[29] Kunisaki SM (2021). Narrative review of congenital lung lesions. Transl Pediatr.

[30] Kane SC, Da Silva CF, Crameri JA, Reidy KL, Kaganov H, Palma-Dias R (2019). Antenatal assessment and postnatal outcome of fetal echogenic lung lesions: a decade's experience at a tertiary referral hospital. J Matern Fetal Neonatal Med Off J Eur Assoc Perinat Med Fed Asia Ocean Perinat Soc Int Soc Perinat Obstet.

[31] Tang S, Meng Q, Chen L, Bekedam H, Evans T, Whitehead M (2008). Tackling the challenges to health equity in China. Lancet.

[32] Pan F, Li J, Lou H, Li J, Jin Y, Wu T (2022). Geographical and socioeconomic factors influence the birth prevalence of congenital heart disease: a population-based cross-sectional study in Eastern China. Curr Probl Cardiol.

[33] Pogoriler J, Swarr D, Kreiger P, Adzick NS, Peranteau W (2019). Congenital cystic lung lesions: redefining the natural distribution of subtypes and assessing the risk of malignancy. Am J Surg Pathol.

[34] Zhang H, Tian J, Chen Z, Ma X, Yu G, Zhang J (2014). Retrospective study of prenatal diagnosed pulmonary sequestration. Pediatr Surg Int.

[35] Stoiber B, Moehrlen U, Kurmanavicius J, Meuli M, Haslinger C, Zimmermann R (2017). Congenital lung lesion: prenatal course, therapy and predictors of perinatal outcome. Ultraschall Med.

[36] Girosi D, Bellodi S, Sabatini F, Rossi GA (2006). The lung and the gut: common origins, close links. Paediatr Respir Rev.

[37] Chen HW, Hsu WM, Lu FL, Chen PC, Jeng SF, Peng SS (2010). Management of congenital cystic adenomatoid malformation and bronchopulmonary sequestration in newborns. Pediatr Neonatol.

[38] Cho MK, Lee MY, Kang J, Kim J, Won HS, Lee PR (2020). Prenatal sonographic markers of the outcome in fetuses with bronchopulmonary sequestration. J Clin Ultrasound JCU.

[39] An P, Wang Y, Feng W, Zhang JQ, Ning YX, Yin JB (2019). Congenital cystic adenomatoid malformation volume ratio in prenatal assessment of prognosis of fetal pulmonary sequestrations. Curr Med Sci.

[40] Van Raemdonck D, De Boeck K, Devlieger H, Demedts M, Moerman P, Coosemans W (2001). Pulmonary sequestration: a comparison between pediatric and adult patients. Eur J Cardio-Thorac Surg Off J Eur Assoc Cardio-Thorac Surg.

[41] Hermelijn SM, Zwartjes RR, Tiddens H, Cochius-den Otter SCM, Reiss IKM, Wijnen RMH (2020). Associated anomalies in congenital lung abnormalities: a 20-year experience. Neonatology.

[42] Conran RM, Stocker JT (1999). Extralobar sequestration with frequently associated congenital cystic adenomatoid malformation, type 2: report of 50 cases. Pediatr Dev Pathol Off J Soc Pediatr Pathol Paediatr Pathol Soc.

[43] Coughlin MA, Gupta VS, Ebanks AH, Harting MT, Lally KP (2021). Incidence and outcomes of patients with congenital diaphragmatic hernia and pulmonary sequestration. J Pediatr Surg.

[44] Gerle RD, Jaretzki A, 3rd, Ashley CA, Berne AS. Congenital bronchopulmonary-foregut malformation. Pulmonary sequestration communicating with the gastrointestinal tract. New Engl J Med. 1968;278(26):1413–9. 10.1056/NEJM196806272782602 5652625

[45] Wong MCY, Faure Bardon V, Farmakis K, Berteloot L, Lapillonne A, Delacourt C (2021). Ultrasound detected prenatal hyperechoic lung lesions and concordance with postnatal findings: a common aspect for multiple diagnoses. Prenat Diagn.

[46] Zobel M, Gologorsky R, Lee H, Vu L (2019). Congenital lung lesions. Semin Pediatr Surg.

